# Role of sex in immune response and epigenetic mechanisms

**DOI:** 10.1186/s13072-024-00525-x

**Published:** 2024-01-22

**Authors:** Sombodhi Bhattacharya, Debasmita Sadhukhan, Radha Saraswathy

**Affiliations:** 1 Biomedical Genetics Research Lab, School of Biosciences and Technology, Vellore Institute of Technology, Vellore, 632014 India; 2 Department of Biomedical Sciences, School of Biosciences and Technology, Vellore Institute of Technology, Vellore, 632014 India

**Keywords:** Sex chromosomes, Immune responses, Epigenetics, Autoimmunity, Cancers immunomodulators

## Abstract

The functioning of the human immune system is highly dependent on the sex of the individual, which comes by virtue of sex chromosomes and hormonal differences. Epigenetic mechanisms such as X chromosome inactivation, mosaicism, skewing, and dimorphism in X chromosome genes and Y chromosome regulatory genes create a sex-based variance in the immune response between males and females. This leads to differential susceptibility in immune-related disorders like infections, autoimmunity, and malignancies. Various naturally available immunomodulators are also available which target immune pathways containing X chromosome genes.

## Background

The immune response in males and females presents numerous differences, affecting the diagnosis, pathogenesis, and treatment of autoimmune diseases and infections. Females generally tend to have a more pronounced immune response, both innate and adaptive, compared to males. The statistics of the recent COVID-19 pandemic showed that although the infection rate varied between males and females the mortality rate and the number of patients admitted to intensive care units (ICUs) were higher in males [24] However, females are more predisposed toward autoimmune diseases. These significant differences in the functioning of the immune system of males and females are due to X and Y sex chromosomes and sex hormones [1].

Males and females have different genetic makeups with males having one X chromosome (XY) and females having two X chromosomes (XX). However, to maintain equivalent gene dosage between the two sexes, one of the X chromosomes in females is inactivated [77]. The X chromosome inactivation happens by epigenetic mechanisms [64] and the inactive X chromosome is associated with repressive epigenetic markers, such as long non-coding RNAs (lncRNA), Firre, XIST, and Dxz4 [28], increased methylation levels [76] and histone modifications. The X chromosome that will be inactivated is chosen at random and the XIST (X inactive specific transcript) RNA determines which X chromosome will be inactivated. However, about 15% of the genes in the inactive X chromosome escape the inactivation process [50]. This may create dimorphisms in immune response as the X chromosome has the highest number of immune-related genes [5, 102]. Sex hormones such as estrogen, progesterone, and testosterone change the immune response as they regulate the cells involved in the immune response [57]. Both the X chromosomes (Paternal and Maternal) have a 50% chance of getting inactivated in females, due to the random nature of the inactivation process, however, one of the chromosomes is more dominant towards inactivation than the other, hence making the inactivation process highly skewed [6]. A higher skewing ratio is typically found in autoimmune disorders [85].

The Y chromosome is known as the functional wasteland as it contains a very low number of genes and is mostly inactive. But some genes of the Y chromosome have regulatory functions; hence they show some sex-dependent dimorphism in the immune response [68].

Immune pathways like TLR 7/8, NF-κB and JAK–STAT have downstream proteins which are encoded in the X chromosome. These escape X inactivation and are highly expressed in autoimmune disorders. Various inhibitors to components of these pathways are listed in Tables 1, 2 and 3. Inflammatory responses in malignancies show difference in immune cell infiltration into tumors like tumor associated neutrophils, macrophages and B cells.

**Table 1:** Naturally available compounds inhibiting the TLR 7 pathway

Compound	Class	Natural sources	Mechanism of action	References
1,4-Naphthoquinone	Quinone	(i) English walnut (*Juglans regia*) (ii) American black walnut (*Juglans nigra)*	Inhibits IRAK 1 and it’s downstream molecules in in vitro human THP-1 macrophages	[52]
Artemisinin derivative SM934	Sesquiterpene lactone	Sweet wormwood (*Artemisia annua*)	Downregulates TLR 7 expression, suppressing B cell activation and Plasma cell formation	[98]
Thiostrepton	Oligopeptide	*Streptomycetes*	Functions as TLR 7 antagonist	[43]
Andrographolide	Diterpenoid	Green Chirata *(Andrographis paniculata)*	Promotes MyD88 degradation	[75]
Azithromycin	Macrolide	*Streptomycetes*	Inhibited proteolytic processing of TLR 7	[43]

**Table 2:** Naturally available compounds inhibiting the NF-κB pathway

Compound name	Class	Natural sources	Mechanism of action	References
β-Cryptoxanthine	Carotenoids	Tangerines, persimmons, oranges	Elevates levels of IgM (B Cell receptor)	[9]
Lycopene	Carotenoids	Tomatoes, grapefruit	Elevated Levels of IgM	[27]
Betulinic acid	Pentacyclic terpenoid	*Betula pendula* (Birch tree) *Eucalyptus* *Platanus* (plane tree)	Increases levels of IgM	[60]
Tanshinone I	Diterpene	*Salvia miltiorrhiza *(red sage)	Syk inhibitor	[95]
Piceatannol	Stilbene	*Vitis vinifera *(red grapes)	Syk inhibitor	[13]
Quercetin	Flavonoid	Onions, apples, grapes	Syk inhibitor	[3]
Curcumin	Diarylheptanoid	*Curcuma longa *(turmeric)	Syk inhibitor, modulates CD 40L expression	[55, 101]
Glabrene	Flavonoid	*Glycyrrhiza glabra* (L*icorice)*	Lyn inhibitor	[34]
Lactucopicrin	Sesquiterpene lactone	*Lactuca virosa* (wild lettuce)	Lyn inhibitor	[34]
Honokiol	Biphenol	*Magnolia *(laurel magnolia)	Lyn inhibitor, inhibiting proliferation and invasion, inducing apoptosis in Adenocarcinoma cells	[21]
Cannabidiol	Resorcinols	*Cannabis sativa *(hemp)	Modulates CD 40L expression	[55]
Azithromycin	Macrolide	*Streptomycetes*	Inhibits CD 40	[43]
Glycyrrhizin	Triterpene saponins	*Glycyrrhiza glabra *(liquorice)	Upregulated CD 40 expression	[7]

**Table 3:** Naturally available compounds inhibiting the JAK/STAT pathway

Compound name	Class	Natural sources	Mechanism of action	References
Duramycin	Thiopeptide	*Streptomycetes*	CXCR 3 inhibitor	[61]
Roselipins	Polyketide glycosides	*Clonostachys rosea*	CXCR 3 inhibitor	[61]
Hypoglausin A	steroidal glycosides	*Blighia sapida *(ackee fruit)	CXCR 3 inhibitor	[61]
Dioscin	Steroidal glycosides	*Scrophularia nodosa L. *(figwort)	CXCR 3 inhibitor	[61]
Gallic acid	Phenolic acid	Blueberry, blackberry, strawberry, plums, grapes, mango, cashew nut, hazelnut, walnut, tea, wine	IL2 inhibitor, FoxP3 inhibitor	[38]

This review attempts to summarize the recent advancements on how sex chromosome epigenetics can affect dimorphisms in the immune responses. It also provides insights on how X chromosome genes can affect the immune pathways and differential responses of infiltrating immune cells in tumors.

## Sex chromosome epigenetics and effects on the immune system

### X chromosome mosaicism, inactivation epigenetics and escape

The paternal X chromosome in females is inactivated to compensate for the dosage of genes between males and females [51]. Phenotypic variations of the male and female immune systems happen due to two processes: X chromosome mosaicism [83] and X chromosome inactivation escape. Ohno and Haushuka reported in 1960 that one X chromosome in females is heteropyknotic [51]. Lyon et al. further proved that one X chromosome is selected randomly at the embryonic stage for inactivation. Lyon used mosaicism to explain the patches of white, normal, and intermediate color in mice with heterozygous sex-linked genes which affected coat color [50]. Some of the autosomal genes determining coat color got translocated on the X chromosome and included a part of linkage group VIII, and the gene for the brown allele translocated on the X chromosome. The patches which were produced by translocated genes showed mosaicism [51]. However, it was also hypothesized that homologous genes to Y should escape inactivation to achieve equivalent gene dosage [10]. Carrel et al. reported that about 15% of genes on the X chromosome could escape the X inactivation, and 10% of these genes are heterozygous, leading to a variation in X-linked gene expression [11].

**Fig. 1:**
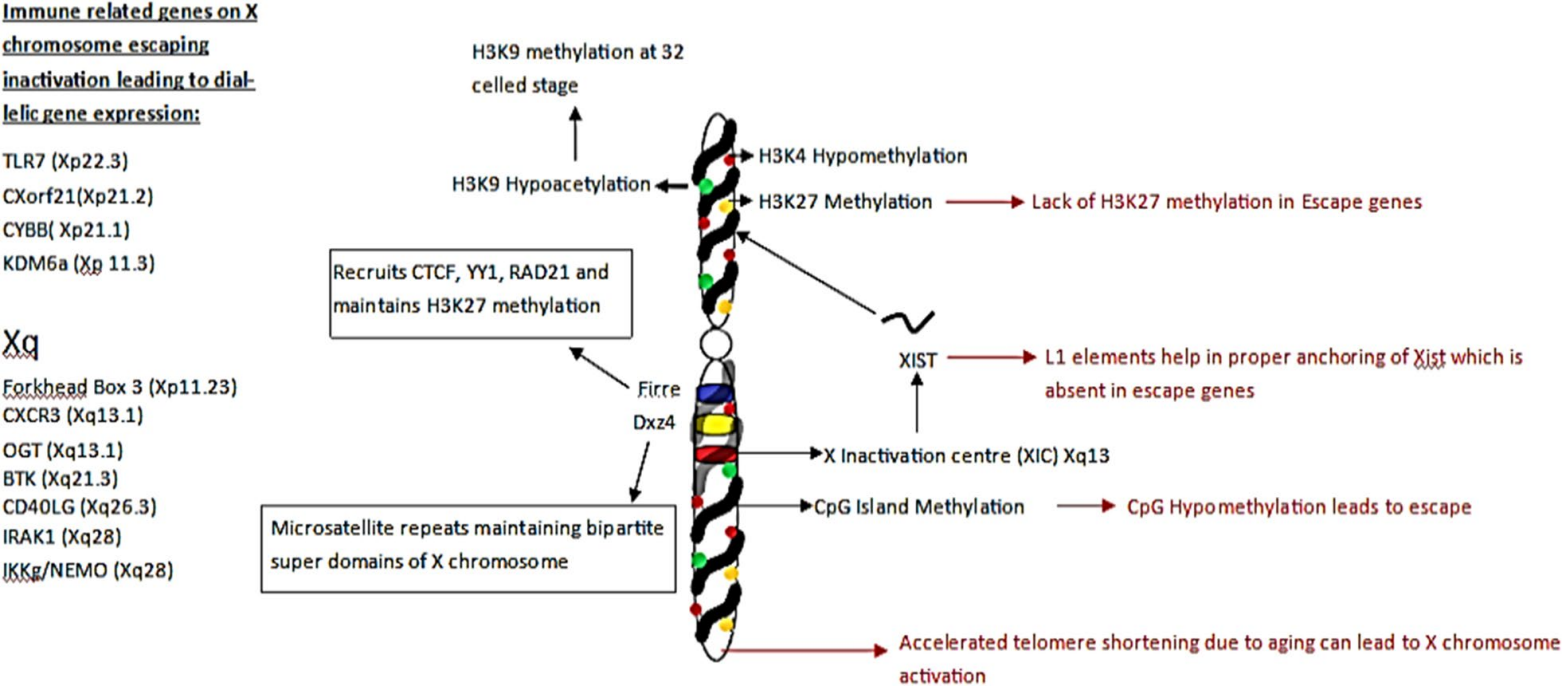
Epigenetic events happening in the human X chromosome and the epigenetics of X chromosome inactivation escape (in red). (On left) genes escaping the X chromosome inactivation process which have implications on the immune system

There are various epigenetic mechanisms which are involved in the inactivation of the X chromosome (Fig. 1). X inactivation happens before implantation and affects numerous epigenetic markers. XIST is a lncRNA that employs a complex responsible for chromosome silencing [4]. XIST RNA expression is seen on embryonic days 2–4 [17]. *Ftx* is a non-coding gene present on the X chromosome inactivation center which acts as a cis-activator of Xist transcription [35]. Dzx4 is another lncRNA associated with X inactivation. It is a microsatellite repeat, maintaining the bipartite super domain of the X chromosome. It is also involved in the formation of chromatin loops by CTCF-mediated interactions, which is responsible for the packaging of the inactive X. Another lncRNA, Firre, is also present, which is involved in recruiting chromatin organizers such as CTCF, YY1, and RAD 21 and maintains H3K27me3 methylation [28]. Inactive X also has inactivating methylation markers hypomethylated H3K4 and hypoacetylated H3K9, which appear at the 8-celled stage. Eed-Ezh2, H3K27 methylation, and macrohistone H2A association occur at the 16-celled morula stage, followed by Histone H3K9 methylation occurring at the 32-celled stage [50].

The region of the X chromosome which escapes the X inactivation is known as the pseudo-autosomal region (PAR), and the genes in this region have homologs on the Y chromosome (Sawalha, Harley, and Scofield 2009). The X-linked genes that escape X inactivation are found in the p-arm of the X chromosome, which may be correlated with its distance from the XIST region.

CpG islands also correlate with escape, as methylation plays a significant role [11]. L1 elements help properly anchor the X chromosome to the XIST lncRNA and are absent in the escape genes. Accelerated telomere shortening due to aging correlates with X chromosome reactivation (XCI) [4]. Liu et al*.* recently reported age-related differential in methylation in regions which escape XCI [49]. Sometimes, if the Y chromosome loses its analog, XCI (X chromosome Inactivation) escape may occur to maintain gene dosage. X chromosome also has an extremely high number of microRNAs. There are only 118 microRNAs on the X chromosome compared to only 4 on Y chromosome—an average of 40–50 microRNAs in the autosomes [22]. These can also escape the meiotic sex chromosome inactivation event as they have a role in meiosis. TSIX is a gene antisense to the XIST gene. Before the inactivation event happens in the embryo, the TSIX expression is higher than XIST. TSIX checks any pre-imprinted bias for inactivation towards one chromosome, leading to an increase in the levels of H3K4me3 on the XIST-associated chromatin and hence an increase in the expression of XIST and Tsix [8]. There are many macro-RNAs present in the inactive X chromosome that govern the decision as to which X chromosome will be inactivated. DXPas34, located at 3’ to XIST, ensures that the X chromosomes remain in the N-1 condition. Xite, a small gene upstream of the Tsix, modulates its expression whose differential methylation patterns along the CTCF (CCCTC binding factor) binding can modulate the Tsix expression at the early paired X stage.

### Inactivation skewing and autoimmune disorders

Theoretically, the X chromosome inactivation happens in a 50:50 manner, however deviations from this make the process of X chromosome inactivation is highly skewed where, generally, the inactivation of one X chromosome is favored over the other [84]. This skewing pattern however shows variations across the body. Zito et al*.* showed that 11% of genes escaping XCI, are constitutively expressed and 23% is tissue restricted [104]. Skewing patterns also show imbalance with age [69]. This has implications in autoimmune diseases. Shvetsova et al. reported in a study that 17.7% of individuals showed skewing towards the maternal X chromosome and 31.6% towards the paternal X chromosome. Seven of the 79 female test candidates showed 100% skewing in all blood cells. No correlation was found between the skewing patterns of mother and daughter, proving that the X inactivation process was random [6]. Chachoub et al. showed that skewing was > 80% in 34.2% of individuals with rheumatoid arthritis and 26% of patients with autoimmune thyroid disease. Mononuclear cells have more skewing due to a higher turnover [14].

Dendritic cells derived from late blastula hematopoietic stem cells will present the self-antigen on only one X chromosome. Hence, T cells interacting with cells containing antigens on the second X chromosome will be directly attacked by cell-mediated immunity. Stewart et al. showed that near 0:100 (only one X chromosome is presented in all the body cells), this would allow some sets of dendritic cells to present an endogenous X antigen and the generation of autoreactive T cells. So, if the skewing is 50:50, taking 15 dendritic cells per T cell, 1 in 10,000 T cells will be autoreactive. Similarly, with a 10:90 skewing, 20% of T cells become autoreactive [4]. Autosomal genes determining coat color are translocated on the X chromosome (used to define mosaicism). Similar translocations may occur in the case of immune-related genes. Changnon et al. showed that in SLE t(X;Y)(p22.33;p11.2), translocation can give rise to duplication of the PAR “Background” (pseudo-autosomal region) region genes. 2 out of the 12 genes held particular interest, IL3, which is a growth factor in HSC (hematopoietic stem cell) development, and CD99, which is involved in the adhesion and apoptosis of T cells.

### Variations in inactivation epigenetics in immune cells

Xist regulation is dynamic in female lymphocytes, which can give rise to the biallelic expression of some X-linked immune genes, such as TLR 7, CXCR 3, and CD 40L. The dynamic nature of Xist RNA expression can be understood by its expression in various sets of lymphocyte cells. It is absent in plasmacytoid dendritic cells but present in the nucleus of natural killer cells and dendritic cells and co-located with H3K27me3 in macrophages made from the bone marrow. T and B cells, both naïve and mature, lack inactivating epigenetic modifications on the inactived X chromosome [17].

Based on the nuclear localization of XIST RNA, Wang et al. classified the Xist RNA expression into four types, based on RNA FISH (fluorescent in situ hybridization) performed by double-stranded exon 1 and short oligos: Type 1, nuclei contain tightly clustered pinpoints localized to one X chromosome; Type 2: diffuse pinpoints encompass the nuclear area and have a size of one whole X chromosome, Type 3 with dispersed pinpoints extending beyond the X chromosome and Type 4 which lacks the Xist expression [92]. Natural killer cells were deficient in detectable Xist RNA signals (Type 4), and only 20–30% had type 3 signals. Myeloid and lymphoid dendritic cells have Type 2 signals. T cells stimulated with CD 3 or CD 28 showed type 1 XIST RNA clouds. COT RNA holes with XIST clouds can detect XIST localization with transcriptional silencing of chromosomes. In this, the naïve T cells showed faint COT 1 signals, and activated T cells showed comparatively more intensity. Type 1 XIST RNA decreased after five days of stimulation, and the number of type 3 levels increased. In macrophages produced by the bone marrow, LPS stimulation increased the Type 1 signals but not type 2 or type 3. Type 1 signal cells were observed to decrease in number after 3–4 days of stimulation. Xist RNA was observed to co-localize with H3K27me3 in 30–50% of cells, but 20–50% of cells lacked the H3K27me3 signal. Xist RNA presence is required for the H3K27me3 signal. No detectable Xist RNA was detected in plasmacytoid dendritic cells, which are type 4 cells. It was not found to be localized in inactivated X chromosomes and lacked H3K27me3 foci, causing more expression of TLR7 in systemic lupus erythematosus related diseases [86].

The X-linked gene dosage in individuals with X-linked lymphoproliferative syndrome who have chronic inflammatory bowel illness XIAP impacts natural killer cell function, highlighting the importance of X-linked gene expression in natural killer cells. It was discovered that, unlike lymphocytes, resting macrophages produced from bone marrow had largely Type 2 Xist RNA patterns and that CpG stimulation produced few Type 1 cells in vitro. As a result, the epigenetic characteristics of Xi in female bone marrow-derived macrophages are more similar to those of female fibroblasts, albeit with weaker Xist RNA clouds. H3K27me3 foci are associated with Xist RNA localization on the inactivated X chromosome. Splenic myeloid and lymphoid dendritic cells exhibit stronger and more detectable Xist RNA signals, with the majority of these dendritic cells classed as Type 3 with Xist RNA distributed across the nucleus and some cells classified as Type 2 with clustered Xist RNA pinpoints. Plasmacytoid dendritic cells are unique because they lack detectable Xist RNA and are only Type 4. However, H3K27me3 foci were seen in 10–20% of plasmacytoid dendritic cells, indicating that Xist RNA localization at the inactivated X chromosome is not needed for H3K27me3 enrichment in these cells. Probably, 80–90% of plasmacytoid dendritic cells that do not have Xist RNA/H3K27me3 enrichment is poised for inactivated X chromosome-mediated gene reactivation. TLR7 expression is biallelic in some plasmacytoid dendritic cells. This suggests that Xist RNA and heterochromatin marks localized on the inactivated X chromosome promote transcriptional silence, and when these epigenetic alterations are lacking, gene reactivation occurs more readily from the Xi. Six X-linked immune genes, including TLR7, were dose compensated in female mouse plasmacytoid dendritic cells. B cells from patients with SLE show reduced XIST RNA and histone H2AK119 mono-ubiquitination (H2AK119Ub) [67].

### Effects of epigenetic inactivation on immune cells

Xist RNA localized to the Xi is not required for X-linked gene dosage compensation in female plasmacytoid dendritic cells. In plasmacytoid dendritic Cells, Xist transcription and localization are uncoupled, which could explain the lack of H3K27me3 accumulation on the inactivated X chromosome in these cells. In female and male plasmacytoid dendritic cells stimulated with R848, there were no significant sex differences in the expression of these genes. In female plasmacytoid dendritic cells, higher levels of IFN production have been found than in male plasmacytoid dendritic cells. While IFN concentrations varied, it was observed that no significant increase in IFN production in female plasmacytoid Dendritic Cells, implying that, unlike humans, female and male plasmacytoid dendritic cells produce equivalent quantities of IFN. No significant differences in *CXCR3, CFP, IRAK1, IL2RG, MSN*, and *TLR7* gene expression were observed between men and women. These findings show that female mouse plasmacytoid dendritic cells can sustain X-linked gene dosage compensation without X-linked gene dosage compensation.

### Dimorphism in immune response due to X chromosome genes

Several X chromosome genes like CD40L, CXCR3, and OGT (O-linked N-acetylglucosamine (GlcNAc) transferase) are upregulated in patients with SLE. Klinefelter’s syndrome patients are likelier to develop female-predominant autoimmune disorders like acquired hypothyroidism, Addison’s disease, and multiple sclerosis In experimental autoimmune encephalitis, autoantigen sensitized XX lymph node was more encephalitogenic than XY. To test this, the effect of Y chromosome genes needed to be removed, which was done by deleting the Sry gene producing an XX and XY-mouse strain. To achieve this, the SJL (Swiss James Lambert) mouse strain, which was chosen due to its greater female-biased disease severity, was chosen. Sry deficient Y chromosome was backcrossed with the original outbred MF1 mice to the SJL until F 16 generation to get XY-mice. These mice were gonadectomized to reduce the effects of the sex chromosome, hence producing pure XXsry and XY^−^sry [8]. Proteolipoprotein (PLP) 139–151 sensitized lymph node cells derived from XX mice and XY^−^ mice were transferred into a common recipient, XX cells have more severe EAE (experimental autoimmune encephalomyelitis) compared to those receiving XY- [25]. Hence, the sex chromosome affects the induction of encephalitogenic immune responses during immunization of adult mice with PLP (phospho lipoprotein) 139–151. Sex chromosomes are expressed more than autosomes in the brain, which may also explain sex bias [25] Pristane was injected into XXsry and XY-sry mice to induce lupus. XX mice showed a greater tubular disease score and chronic lesions compared to XY-. XX mice also showed higher levels of IgG anti-ds DNA antibodies when compared to XY-mice. The survival rate was also significantly lesser in XY-mice with 30.4% compared to 68.4% in the XY-mice. This was even seen in gonad-intact mice, with a 27.3% survival rate in XX mice compared to 66.7% in XY-mice. PLP-immunized mouse lymph node cells (LNCs) were drained to assess for cytokine production, and it was found that Th2 cytokines, IL 1, and IL 15 were higher in XY-mice than XX. Pristane-injected mice and anti-CD 3 and CD 28 cultured splenocytes showed higher levels of IL 13 and IL 5 in XY-mice. IL13Rα is a decoy receptor with Th2 suppressing activity detected in dendritic cells, macrophages, and B cells in spleens of PLP autoantigen immunized mice with a higher expression in XX mice [80].

## Y chromosome and implications on the immune system

The Y chromosome is one of the shortest chromosomes in the human genome, containing fewer protein-coding genes [79]. It is heterochromatic, possesses only a few sex-determining genes, and is mainly composed of multicopy genes, repeat sequences, and transposable elements [103]. The Y chromosome contains 64 protein-coding, 45 male-specific sequences, and 19 pseudo-autosomal region genes. Due to these attributes, it is usually known as the functional wasteland [8]. But this chromosome is actively involved in gene regulation by affecting chromatin dynamics through its variation in multicopy ribosomal genes [103].

There are some reports of the implications of this chromosome on the immune system mostly because of its regulatory functions. Kutch et al. showed that Y-linked regulatory variations have effects on the IMD pathway, which is highly conserved as an NF-kB immune pathway in Drosophila [58]. RT-PCR analysis of three genes in the pathway showed two genes had variation in expression: attacin A and cecropin A 1. The CT values of all three genes were positively correlated. Case et al. showed that genetic variations in the Y chromosome affected the susceptibility of B6 Y consomic mice to experimental autoimmune encephalomyelitis and showed dimorphism. The basal levels of natural killer T cells were also affected by gene variations outside the sex-determining region of the Y chromosome [12]. A mosaic loss of Y chromosomes (LOYs) is also found in the leukocytes of male individuals, which can affect the immune system. Mattison et al. showed that single cells with LOY showed a decreased expression of CD 99 mRNA and surface protein, which was evaluated by the CITEseq assay. For this, cells were treated with oligonucleotide conjugated CD 99 antibodies to have a barcode similar to the scRNA seq. LOY status was examined using the male-specific region of the Y chromosome. LOY frequency was found in 2.4–18.3% in all cell types [54].

Dumanski et al. showed that the loss of Y chromosomes in immune cells dysregulated the autosomal genes. Loss of Y chromosomes was tested in peripheral blood mononuclear cells (PBMCs) from 29 males, and single-celled transcriptome sequencing was done (scRNAseq). LOYs were detected in all 29 males but varied between all cell types: NK cells, monocytes, B and T lymphocytes were 27% (7–87%), 23% (7–87%), 7% (2–40%), and 3% (1–6%), respectively**.** Pairwise samples studied In vivo with scRNAseq, RNAseq, and DNAseq showed highly consistent LOY as well as in vitro studies with 13 lymphoblastoid cell lines, which were done by an SNP-based array using digital droplet PCR targeting a 6bp sequence difference between AMELY and AMELX. LOY-associated transcriptional effects (LATE) were tested between LOY and non-Loy cells. It was seen that MSY genes had reduced expression in the bulk RNA seq and were absent in the single-celled RNA seq. The PAR genes also showed decreased expression, however not as distinct as that of MSY genes, as they are even expressed in the PAR region of the X chromosome. In the case of autosomes, 489 LATE genes were found, and ten non-PAR LATE X chromosome genes were found, which showed both over and under-expression, with LYPD2 showing 8.6-fold higher and IL2R showing 2.5 times lower. Autosomal genes expressed with MSY also showed differential expression patterns in single cells with LOY [26].

## Immune pathways of X chromosome genes and natural immunomodulators

**Fig. 2:**
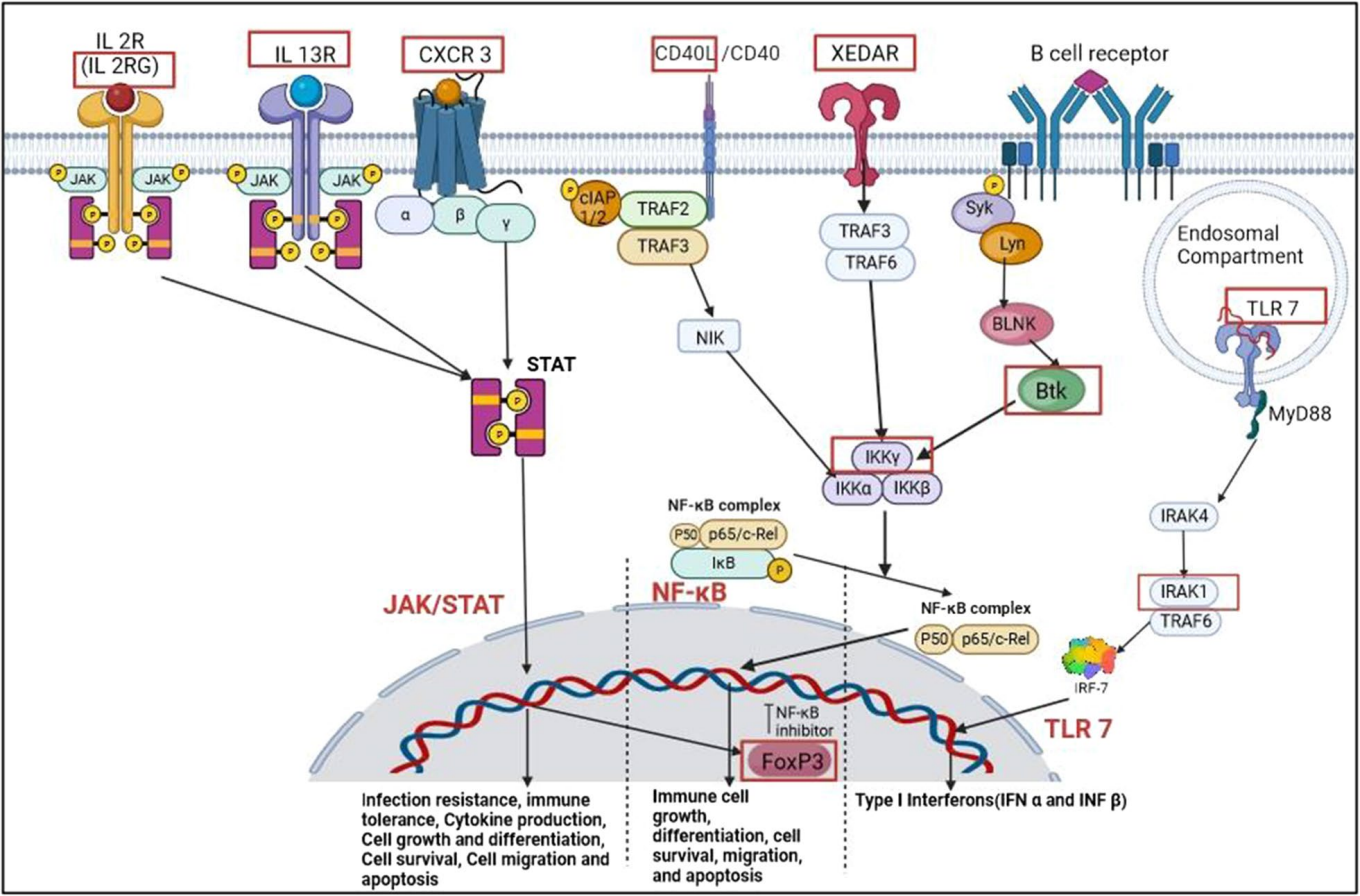
Immune pathways related to X chromosome genes (X chromosome genes are in red boxes)

The X chromosome has the highest density of immune-related genes involved in innate and adaptive immune responses [73]. Various pathways significant to the functioning of the immune system have genes which are located on the X chromosome, have been explained in this section. Natural immunomodulators specific to these pathways have also been tabulated (Tables 1, 2, 3). Figure 2 shows all the pathways in the immune system which involve X chromosome genes.

### Toll like receptor 7 (TLR 7) pathway

Toll like receptor 7 (TLR 7) is an intracellular immune sensor present on the endosomal compartment, which recognizes ss-RNA containing immune complexes compartment [65]. The gene encoding *TLR 7* is present on the q-arm of the X chromosome (Xq22.3) [29]. TLR 7 upon activation recruits toll interleukin receptor (TIR) containing adaptor, My D88 which then forms a complex with a group of Interleukin-1 receptor associated kinases (IRAKs), forming a Myddosome. IRAK 4 activates IRAK 1 which then associates with tumor necrosis factor receptor associated factor (TRAF 6), a RING-domain associated E3 domain associated ligase. The *IRAK-1* gene is present on the q-arm of the X chromosome (Xq28) [39]. TRAF-6 then activates Interferon Regulatory factor 7 (IRF-7) [42] which leads to the transcription of Type 1 interferons (IFN α and INF β) [36] hence activating an innate immune response. TLR 7 is mostly expressed in B cells and plasmacytoid dendritic cells (pDCs).

TLR 7 is associated with systemic lupus erythematosus (SLE) [93]. Souyris et al. [81] assessed the escape of this gene is SLE patients utilizing the detection of diallelic single nucleotide polymorphisms (SNPs) located on the 5’ untranslated region (UTRs) of the TLR 7 mRNA. In 40% of B cells, 23.8% monocytes and 18.5% showed biallelic tags showing X inactivation escape. A fraction of CD27 + B cells displayed two nuclear foci on RNA FISH experiments, one was located on the inactive X chromosome, which was found by XIST non-coding region-specific probe. Resting PBMCs (peripheral blood mononuclear cells) showed a 1.31- to 1.38-fold increase in the expression of TLR 7 in resting PBMCs. The CD 27 + plasma cells get activated and expand more vigorously in females than in males on stimulation with TLR 7 activator IFN I (Interferon I). Increased expression of TLR 7 also increases the frequency of IgG class switching, hence inactivation escape may lead to greater class switching in B cells. TLR 7 inactivation escape was also reported in males with Klinefelter’s syndrome, which elevated their risk of developing SLE. Claudia et al. 2023 also showed an increase in the number of IRF-5 positive B cells on stimulation of TLR 7. *IRAK 1* also escapes X chromosome inactivation and hence making the TLR signaling pathway more pronounced in females as compared to males. Spiering and Vries [82] reported that a pronounced TLR 7 signaling has led to the sex-based bias in COVID 19 infections and death rates.

Table 1 shows the naturally available immunomodulators which acts on the TLR 7 pathway.

### Nuclear factor kappa light chain enhancer of activated B cells (NF-κB) pathway

NF-κB is a transcription factor responsible for multiple aspects of the innate as well as adaptive immune system. It transcribes genes for pro-inflammatory cytokines and chemokines, for inflammasome regulation as well as genes responsible for immune cell growth, differentiation, cell survival, migration, and apoptosis [48]. NF-κB transcription factor is activated by several pathways of the immune system and the pathways include X chromosome genes.

The NF-κB transcription factor is bound to the inhibitor of κB proteins (IκB), and for its activation IκB has to be phosphorylated after which NF-κB is disassociates from IκB. IκB is phosphorylated by IκB kinase complex (IKK), which is a complex formed by 3 kinases: IKKα, IKKβ and NF-κB essential modulator (NEMO). NEMO is encoded by the *Inhibitor Of Nuclear Factor Kappa B Kinase Regulatory Subunit Gamma (IKBKG)* gene which is present on the X chromosome (Xq28) [41]. The NEMO gene is central to canonical NF-κB activation [87]. Hence all types of canonical activators of NF-κB will be involve NEMO. Pattern recognition receptors (PRRs), Tumor necrosis factor receptor (TNFR), B cell and T cell receptors activate the canonical NF-κB pathway [48]. The *NEMO* gene was reported to show heterogeneity in X chromosome escape [62].

There are numerous signaling pathways which activate NF-κB, and these pathways also contain X chromosome genes. The B cell receptor (BCR) activates NF-κB by the canonical pathway for differentiation, proliferation and survival of B cells [36]. BCR activates tyrosine kinases Spleen tyrosine kinase (Syk) and Lck/Yes tyrosine kinase (Lyn) [44], which activates Bruton’s tyrosine kinase (Btk) mediated by B cell linker protein (BLNK). *Btk* gene is located on the X chromosome (Xq21.3-q22). Btk associates with IκB-α and phosphorylates it which activates the NF-κB pathway [66]. The *Btk* gene has been reported to escape X chromosome inactivation in the immune cells of females [33].

The X-linked ectodermal dysplasia receptor (XEDAR) is a recently discovered protein which belongs to the TNF class of receptors. It is an evolutionary conserved pathway, which is involved in the development of ectodermal appendage organs like hair, eccrine sweat glands and mammary glands [97]. XEDAR activates both the canonical as well as non-canonical NF-κB pathway. In the canonical pathway, XEDAR activates the IKK complex via TRAF-3 and -6 [78]. In the non-canonical NF-κB activation, the p100 processing requires TRAF-3 and -6 as well as IKKα, which then processes p100 into p52 which associates with RelB and translocates into the nucleus [90].

The gene for the ligand of cluster of differentiation CD-40, CD 40L/ CD 154, is present on the X chromosome (Xq 26.3). CD 40L activates CD 40, which activates the non-canonical NF-κB pathway. A complex consisting of TRAF-2, -3, cIAP1/2 and NIK is present in the cytoplasm, where the NIK levels are kept low by ubiquitination by TRAF3. On activation, TRAF3 degrades and NIK levels are stabilized and translocate to the cytoplasmic part of the CD 40 receptor. NIK activates IKKα, which then phosphorylates p50 [23] found an increase in CD 40 as well as CD 40L expression on draining lymph node (dLN) T cells and B cells in female adjuvant induced arthritis rats as compared to male rats.

There are numerous natural immunomodulators which act on the mentioned pathway.

### Janus kinase/signal transducers and activator of transcription (Jak/STAT pathway)

Jak/STAT pathway plays a very important role in the functioning of the immune system. It is involved in infection resistance, immune tolerance, cytokine production, cell growth and differentiation, cell survival, cell migration and apoptosis. Aberrant Jak/STAT pathway can also lead to autoimmune diseases [91]. Numerous immune receptors activate the Jak–STAT pathway, which contains X chromosome genes.

Chemokine receptor 3 (CXCR 3) is a chemokine receptor involved in the T cell migration and trafficking. The gene for *CXCR3* islocated on the X chromosome (Xq13.1). CXCR 3 is a G protein coupled receptor, which is activated by chemokine ligands (CXCL), 9, 10 and 11. CXCR 3 can activate the JAK/STAT pathway, and also the PI3K/Akt and Ras/ERK pathway [20]. *CXCR 3* gene has been reported to escape the X chromosome inactivation process and give a sex associated bias in infections [59]. IL 13 receptor (IL 13 Rα and IL 13 Rβ) are encoded on the X chromosome and it activates JAK/STAT pathway. However, IL 13Rα1 is subject to X chromosome inactivation. The gene for the gamma chain of IL 2 receptor, *IL2RG* is expressed on the X chromosome Xq13.1. The IL 2 receptor also activates the JAK-Stat Pathway. IL2RG is involved in X-linked severe combined immunodeficiency (XSCID) [47]. Forkhead Box P3 gene (*FoxP3*) is expressed on the X chromosome (Xp11.23), and is a result of the IL 2 Jak/Stat pathway [30]. FoxP3 suppresses NF-κB and is involved in the suppression of antigen priming of T cells and is involved in maintaining immune tolerance. However, FoxP3 is subjected to X chromosome inactivation [89].

## Sex difference in immune responses and cancers

Cancers are one of the leading causes of mortality in the world causing 1 in 6 deaths globally. Cancers are associated with the immune system. Cancer is associated with inflammatory responses, which is considered as a hallmark for cancer [19]. When tumorigenesis is initiated, inflammation occurs at the tumor site and the immune cells like CD 8 + and CD 4 + T cells, cytotoxic macrophages and neutrophils are activated. However, as the tumor progresses and becomes metastatic, it is able to escape immune recognition. This balance between immune inflammation and immune tolerance, helps the cancer cells to resist the immune system [31].

Li et al. [45] analyzed somatic mutations and mRNA expression in Lung Adenocarcinoma data and found that females had a higher expression of immune-related genes. Male patients with mutations in epidermal growth factor receptor (EGFR) had increased infiltration of CD8 + cells and CD4 + cells in females [45]. Warde et al. [96] showed that males with Zinc finger ring 3 (ZNFR 3) deletion show a greater myeloid cell accumulation and a greater anti-tumor immune response. Neutrophils are affects by male and female sex hormones, with female hormones delaying neutrophil apoptosis and male hormones increasing the neutrophil activation in non-inflammatory states [37]. Hence females have higher neutrophil counts and neutrophil-to-lymphocyte ratio (NLR) females having late menopause have been reported to have a lower risk of developing gastric cancer whereas [63], androgen ablation reduces neutrophils [37]. Tumor associated neutrophils (TAN) were found to be an independent predictor of tumor associated survival rates [18]. TAN was also found to be higher in males in Hepatocellular carcinoma models (HCC) of zebrafishes [99] and in oral cancers [72]. Gwak et al. 2007 reported that females showed more immune compromised response to gastrectomy when compared to male patients and females had a higher neutrophil to lymphocyte ratio (NLR) [32]. A higher NLR value was also reported by Lin et al. 2018 in head and neck squamous cell carcinoma (HNSCC) [46].

Human macrophages express receptors for sex hormones like Progesterone receptor (PR), estrogen receptor (ER) and androgen receptors (AR) and are also subject to hormonal modulation effects [74]. Tumor associated macrophages (TAMs) play an important role in cancer-induced inflammation. TAM number was reported to significantly lower in females than in males in pancreatic ductal adenocarcinoma (PDAC) [53]. However, TAMs form female patients with non-small cell lung cancer, were reported to be more immunogenic and male TAMs were more immunosuppressive with upregulated PPARs and matrix remodeling pathways [100]. Non-muscle invasive bladder cancer (NMIBC) tumors from females showed higher infiltration from M-2 macrophages [16]. Dendritic cells (DCs) infiltrate tumors, however, do not contain any receptors for sex hormones. DC infiltration was reported to be more in female tumors as compared to male tumors [88]. B cells express androgen receptors at pro-B and pre-B stages and hence have an impact on the maturation of B cells. Estrogen controls B cell differentiation, activity and function and has ER-β receptors. Female tumors from NMIBC show increased B cell recruitment.

## Discussion

The twenty-first century is the era of personalized medicine and therapeutics tailored to the patient and one of the primary considerations the is sex of the patient. Biological differences should be taken into consideration while research as well as therapeutics [56]. The immune systems show sex-based dimorphisms resulting in females with immune regulatory genes on the X chromosome able to cope with many illnesses, whereas males are unable to survive. The basic mechanism in the human immune system exhibits sexual dimorphism with immune responses differing between females and males, which is due to various factors like, including sex hormones, fetal microchimerism, sex chromosome abnormalities, and epigenetic mechanisms like skewed X chromosome inactivation. This has important roles in a plethora of diseases like infections, autoimmunity and malignancies.

Maintenance of gene dosage between males and females requires one X chromosome to be silenced, which is achieved by various epigenetic mechanisms like association with lncRNA, hypomethylation and silencing histone modifications. However, this process is imperfect which leads to skewing in the inactivation process and escape of genes from the inactive X chromosome hence, creating dimorphic gene expression patterns between males and females. Skewed inactivation and inactivation escape is increased in autoimmune disorders. Omics approaches can be used to analyze differentially expressed X chromosome genes, in immune-related diseases. Sauteraud et al*.* developed an R package which is able to analyze escape genes from RNA-seq data [70]. Keniry et al*.* have reviewed the findings produced by single-celled genomics approaches to understand inactivation epigenetics [40].

There are many important immune-related genes on the X chromosome which escape the X inactivation event. These genes are part of important immune regulatory pathways like NF-κB and JAK–STAT, which are important for immune responses. Further work needs to be done on how the X chromosome genes affect these pathways and how it can produce a differential immune response in various scenarios. There is a growing interest in phytochemicals and natural compounds with immunomodulatory effect for preventing and managing diseases as they provide complimentary and or alternative drugs. They can also serve as starting points for drug development [13]. There are numerous naturally available compounds which act as inhibitors to the previously mentioned immune pathways which are listed in Tables 1, 2 and 3. Any compound inhibiting proteins coded by X chromosome in the immune pathway as well as inhibiting the proteins preceding will have dimorphisms in action due to the differential expression. However, further work needs to be done to prove the effects of inhibiting X chromosome genes.

The immune system is also related to cancer as malignant tumors can create an inflammatory microenvironment. Various sets of innate immune cells like neutrophils, macrophages and adaptive immune cells like B cells show differences in tumor infiltration. This effect may be due to sex hormones like androgen, progesterone and estrogen as well as due to X chromosome genes. However, further work needs to be done to establish mechanisms behind these sex-based differences in tumor infiltration. Immunotherapy promises an effective solution to cancer therapeutics; however, sex-based differences are reported. Immune checkpoint inhibitors (ICIs) have been reported to be more effective in males as compared to females. This may be due to increased antigenicity in male tumors. Chimeric antigen receptor T cell (CAR-T cell) therapy is coming up as a novel therapeutic solution to cancers. It utilizes the autologous T cells isolated from the patient’s blood followed by expression of a Chimeric antigen receptor specific for the patient’s tumor and injecting it back into the patient body. Sex based differences due to hormones, X chromosome genes, and differences in tumor antigenicity will have an effect on the outcome of the treatment, however further work needs to be done to determine how sex can affect therapeutic outcomes in CAR-T cell therapy.

## Data Availability

Not applicable.

## Abbreviations

lncRNAs: Long non-coding RNAs
XIST: X inactive specific transcript
CTCF: CCCTC binding factor
miRNA: Micro-RNAs
BTK: Bruton tyrosine kinase
IRAK1: Interleukin 1 receptor associated kinase 1
IKKg/NEMO: NF-kappa-B essential modulator
TLR: Toll like receptor
CD: Cluster of differentiation
10.CXCR: Chemokine receptor
TLR: Toll like receptor
SLE: Systemic lupus erythematosus
pDCs: Plasmacytoid dendritic cells
SNPs: Single nucleotide polymorphisms
PBMC: Peripheral blood mononuclear cells
HSC: Hematopoietic stem cell
NK: Natural killer
XCI: X chromosome inactivation
PAR: Pseudoautosomal region
HSC: Hematopoietic stem cell
OGT: O-Linked N-acetylglucosamine (GlcNAc) transferase
PLP: Proteolipoprotein
FISH: Fluorescent in situ hybridization
EAE: Experimental autoimmune encephalomyelitis
CTCF: CCCTC-binding factor
YY1: Ying Yang 1
RAD 21: Double strand break repair protein
IFN: Interferon
CFP: Complement factor properdin
MSN: Moesin
XIAP: X-linked inhibitor of apoptosis of protein
LOYs: Loss of Y chromosomes
TIR: Toll interleukin receptor
TRAF: Tumor necrosis factor receptor associated factor
IRF: Interleukin regulatory factor
IFN: Interferon
Ig: Immunoglobulin
NF-κB: Nuclear factor kappa light chain enhancer of activated B cells
NEMO: NF-κB essential modulator
IKBKG: Inhibitor of nuclear factor kappa B kinase regulatory subunit gamma
PPR: Pattern recognition receptor
TNFR: Tumor necrosis factor Receptor
Syk: Spleen tyrosine kinase
Lyn: Lck/Yes tyrosine kinase
BCR: B cell receptor
BLNK: Btk mediated by B cell linker protein
XEDAR: X-linked ectodermal dysplasia receptor
Jak/STAT: Janus kinase/signal transducers and activator of transcription
CXCR: Chemokine receptor
IL: Interleukin
FoxP3: Forkhead box P3
EGFR: Epidermal growth factor receptor
ZNFR: Zinc finger ring
NLR: Neutrophil-to-lymphocyte ratio
TAN: Tumor associated neutrophils
PR: Progesterone receptor
ER: Estrogen receptor
AR: Androgen receptor
TAM: Tumor associated macrophages
